# 5-oxoprolinuria (Pyroglutamic Aciduria) and Metabolic Acidosis: Unraveling the Mystery

**DOI:** 10.5005/jp-journals-10071-23211

**Published:** 2019-07

**Authors:** Subramanian Senthilkumaran, Florence Benita, Narendra Nath Jena, Sambathkumar Sasikumar, Ponniah Thirumalaikolundusubramanian

**Affiliations:** 1 Department of Emergency and Critical Care, Manian Medical Center, Erode, Tamil Nadu, India; 2 Department of Emergency Medicine, DM Wayanad Institute of Medical Sciences, Wayanad, Kerala, India; 3 Department of Emergency Medicine, Meenakshi Mission Hospital and Research Centre, Madurai, Tamil Nadu, India; 4 Department of Emergency Medicine, Fortis Malar Hospital, Adyar, Chennai, Tamil Nadu, India; 5 Department of Internal Medicine, Chennai Medical College Hospital and Research Center, Irungalur, Trichy, India

**Keywords:** 5-oxoprolinuria, Metabolic acidosis, N-acetyl cysteine, Paracetamol therapy, Recovery, Risk/precipitating factors

## Abstract

A case of high anion gap metabolic acidosis (HAGMA) and high level of 5-oxoprolinuria were noticed in an elderly female of 66 years who had multiple risk/precipitating factors and recovered well with N-acetyl cysteine infusion. This is reported in view of its rarity and to create awareness of this entity among medical students and practicing physicians who handles such cases in emergency room or critical care unit. Moreover they have to remember and investigate the cases of metabolic acidosis for 5-oxoprolinuia especially in susceptible individuals who are on paracetamol with or without other precipitating factors.

**How to cite this article:**

Senthilkumaran S, Benita F, Jena NN, Sasikumar S, Thirumalaikolundusubramanian P. 5-oxoprolinuria (Pyroglutamic Aciduria) and Metabolic Acidosis: Unraveling the Mystery. Indian J Crit Care Med 2019;23(7):342–343.

## INTRODUCTION

Normal acid-base homeostasis is essential for healthy living. Acid-base abnormalities are a frequently seen in emergency settings. Elevated anion gap acidosis represents accumulation of acid anion from organic/inorganic acids. It is commonly caused by lactic acidosis, ketoacidosis, and ingestion of acid-generating poisons, including methanol, salicylates, ethylene glycol etc., Although many ‘classic’ causes of metabolic acidosis are known, γ-glutamyl cycle enzyme defect resulting in accumulation of 5-oxoproline (pyroglutamic acid),^1^ presenting as high anion gap metabolic acidosis (HAGMA) is rarely seen. We present a case of profound metabolic acidosis with raised anion gap and 5-oxoprolinuria in an elderly lady who had multiple risk factors.

## CASE REPORT

A 66-years-old female without any comorbid illnesses was transferred to the intensive care unit from the floor on tenth day of her admission for shortness of breath and declining levels of consciousness. She was initially admitted for cellulites of right leg due to methicillin-sensitive Staphylococcus aureus and treated with flucloxacillin (6 g/day) and acetaminophen 3 g/day.

On examination, she was thin built (BMI 16.5), fragile, drowsy, disoriented, afebrile, mildly dehydrated and under nourished. Her heart rate was 95 beats/minute; blood pressure was 110/70 mm Hg with respiratory rate of 36 breaths/minute and saturation of 98% with 2 L of oxygen by nasal prongs. Chest auscultation did not reveal any abnormalities. Her cardiovascular and abdominal examinations were unremarkable. Though there was no focal neurologic deficit, her Glasgow Coma Scale score was 12 (E 3, V 4, M 5) and pupils were equal (3 mm) and reactive to light.

Arterial blood gas analysis revealed metabolic acidosis with pH 7.22, pO_2_ 91 mm Hg, pCO_2_ 21 mm Hg, bicarbonate 9.9 mmol/L, base excess −16.7, sodium 145 mEq/L, potassium 4.1mEq/L and Chloride 102 mEq/L. Her serum creatinine (1.2 mg/dL), serum lactate (1.6 mmol/L) and plasma sugar (102 mg/dL) were well within normal limits, and could not explain the wide anion gap of 34 mEq/L (Corrected Anion Gap for Albumin 43.3 mEq/L). Her measured serum osmolality was 303 mmol/kg with an osmolar gap of 5.3 mmol/kg. Her Cockcroft-Gault clearance was 35 mL/minute. Urinalysis and urine toxicology testing did not show any evidence of infections and ingestion of chemicals respectively. There was no ketonuria. A computed tomographic scan of brain and high-resolution computed tomography of chest were normal. Her electrocardiography revealed normal sinus rhythm.

Google search led us to the possibility of pyroglutamic acid as a cause of high anion gap metabolic acidosis and hence flucloxacillin and acetaminophen were stopped immediately. She was evaluated for 5-oxoprolinuria and it was found to be 1292 mmol/mmol creatinine, She was treated with bicarbonate and N-acetyl cysteine infusion. Her metabolic acidosis resolved and general condition improved rapidly.

## DISCUSSION

Accumulation of 5-oxoproline (pyroglutamic acid) is a rare cause of high anion gap metabolic acidosis (HAGMA). It was described more in pediatric literature and results from autosomal recessive deficiencies of glutathione synthetase or 5-oxoprolinase. Of late, adult cases are reported.^2^ Glutathione is a tripepide consists of glutamate, cysteine and glycine. It is found in most cell types and utilized in scavenging of free radicals, redox reactions, and formation of deoxyribonucleotidases, leukotrine biosynthesis and amino acid transport. It also provides negative feedback on the gamma glutamyl cycle by inhibiting the enzyme gamma-glutamyl-cysteine synthase. Whenever there is inhibition or deficiency of the enzyme, gamma glutamyl cysteine is converted to 5-oxoproline. An acquired deficiency of glutathione secondary to paracetamol ingestion results in loss of this negative feedback and enhances the production of 5-oxoproline leading to a metabolic acidosis^3^ and 5-oxoprolinuria.

Although there are reports of metabolic acidosis with raised anion gap with acute paracetamol overdose, the majority of the published case reports indicate that these patients received only therapeutic doses of paracetamol for pain or pyrexia. These cases of metabolic acidosis were often associated with acetaminophen in susceptible individuals who had one or other risk factors a combination of many such as age, female gender, vegetarian, pregnancy, malnutrition, sepsis, underlying liver disease, or renal failure^4^ and medications such as vigabatrin, netilmicin and flucloxacillin.

Diagnosis of 5-oxoprolinuria in cases of metabolic acidosis is based on clinical suspicion. Due to non-availability of this investigation in many places and suboptimal awareness of this entity limit its diagnostic utility to physicians. However, it is important for the clinicians to recognize and rule out the common causes of metabolic acidosis as considered in the mnemonic “MUDPILES” (Methanol, Uremia, Diabetes, Paraldehyde, Iron (and Isoniazid), Lactate, Ethylene glycol, and Salicylate). Mehta et al.^5^ had proposed new mnemonic to remember the causes for anion gap “GOLD MARK” (Glycols (ethylene and propylene), Oxoproline, L-lactate, D-lactate, Methanol, Aspirin, Renal failure, and Ketoacidosis) for the 21st century.

To conclude, when the lactate and ketone levels don't correlate with the anion gap in cases of metabolic acidosis without significant history of ingestion of toxic materials, 5-oxoprolinuria shall be considered in patients who have one or other risk factors and with or without the history of recent acetaminophen consumption. Accordingly from the point of patient safety, medical students and practitioners shall be oriented to evaluate such cases for 5-oxoprolinemia or 5-oxoprolinuria as this entity is under appreciated and rarely discussed in clinical rounds.
